# Screening PubMed abstracts: is class imbalance always a challenge to machine learning?

**DOI:** 10.1186/s13643-019-1245-8

**Published:** 2019-12-06

**Authors:** Corrado Lanera, Paola Berchialla, Abhinav Sharma, Clara Minto, Dario Gregori, Ileana Baldi

**Affiliations:** 1 Unit of Biostatistics, Epidemiology and Public Health, Department of Cardiac Thoracic Vascular Sciences and Public Health, University of Padova, Via Loredan, 18, 35131 Padova, Italy; 2 Department of Clinical and Biological Sciences, University of Torino, Torino, Italy; 3 Department of Biological Sciences and Bioengineering, Indian Institute of Technology Kanpur, Kanpur, India

**Keywords:** Classification, Indexed search engine, Machine learning, Text mining, Unbalanced data, systematic review

## Abstract

**Background:**

The growing number of medical literature and textual data in online repositories led to an exponential increase in the workload of researchers involved in citation screening for systematic reviews. This work aims to combine machine learning techniques and data preprocessing for class imbalance to identify the outperforming strategy to screen articles in PubMed for inclusion in systematic reviews.

**Methods:**

We trained four binary text classifiers (support vector machines, k-nearest neighbor, random forest, and elastic-net regularized generalized linear models) in combination with four techniques for class imbalance: random undersampling and oversampling with 50:50 and 35:65 positive to negative class ratios and none as a benchmark. We used textual data of 14 systematic reviews as case studies. Difference between cross-validated area under the receiver operating characteristic curve (AUC-ROC) for machine learning techniques with and without preprocessing (delta AUC) was estimated within each systematic review, separately for each classifier. Meta-analytic fixed-effect models were used to pool delta AUCs separately by classifier and strategy.

**Results:**

Cross-validated AUC-ROC for machine learning techniques (excluding k-nearest neighbor) without preprocessing was prevalently above 90%. Except for k-nearest neighbor, machine learning techniques achieved the best improvement in conjunction with random oversampling 50:50 and random undersampling 35:65.

**Conclusions:**

Resampling techniques slightly improved the performance of the investigated machine learning techniques. From a computational perspective, random undersampling 35:65 may be preferred.

## Background

The growing number of medical literature and textual data in online repositories led to an exponential increase in the workload of researchers involved in citation screening for systematic reviews (SRs). The use of text mining (TM) tools and machine learning techniques (MLT) to aid citation screening is becoming an increasingly popular approach to reduce human burden and increase efficiency to complete SRs [1–6].

Thanks to its 28 million citations, PubMed is the most prominent free online source for biomedical literature, continuously updated and organized in a hierarchical structure that facilitates article identification [7]. When searching through PubMed by using keyword queries, researchers usually retrieve a minimal number of papers relevant to the review question and a higher number of irrelevant papers. In such a situation of imbalance, most common machine learning classifiers, used to differentiate relevant and irrelevant texts without human assistance, are biased towards the majority class and perform poorly on the minority one [8, 9]. Mainly, three sets of different approaches can be applied to deal with imbalance [9]. The first is the pre-processing data approach. With this approach, either majority class samples are removed (i.e., undersampling techniques), or minority class samples are added (i.e., oversampling techniques), to make the data more balanced before the application of an MLT [8, 10]. The second type of approaches is represented by the set of algorithmic ones, which foresee cost-sensitive classification, i.e., they put a penalty to cases misclassified in the minority class, this with the aim to balance the weight of false positive and false negative errors on the overall accuracy [11]. Third approaches are represented by the set of ensemble methods, which apply to boosting and bagging classifiers both resampling techniques and penalties for misclassification of cases in the minority class [12, 13].

This study examines to which extent class imbalance challenges the performance of four traditional MLTs for automatic binary text classification (i.e., relevant vs irrelevant to a review question) of PubMed abstracts. Moreover, the study investigates whether the considered balancing techniques may be recommended to increase MLTs accuracy in the presence of class imbalance.

## Methods

### Data used

**Fig. 1:**
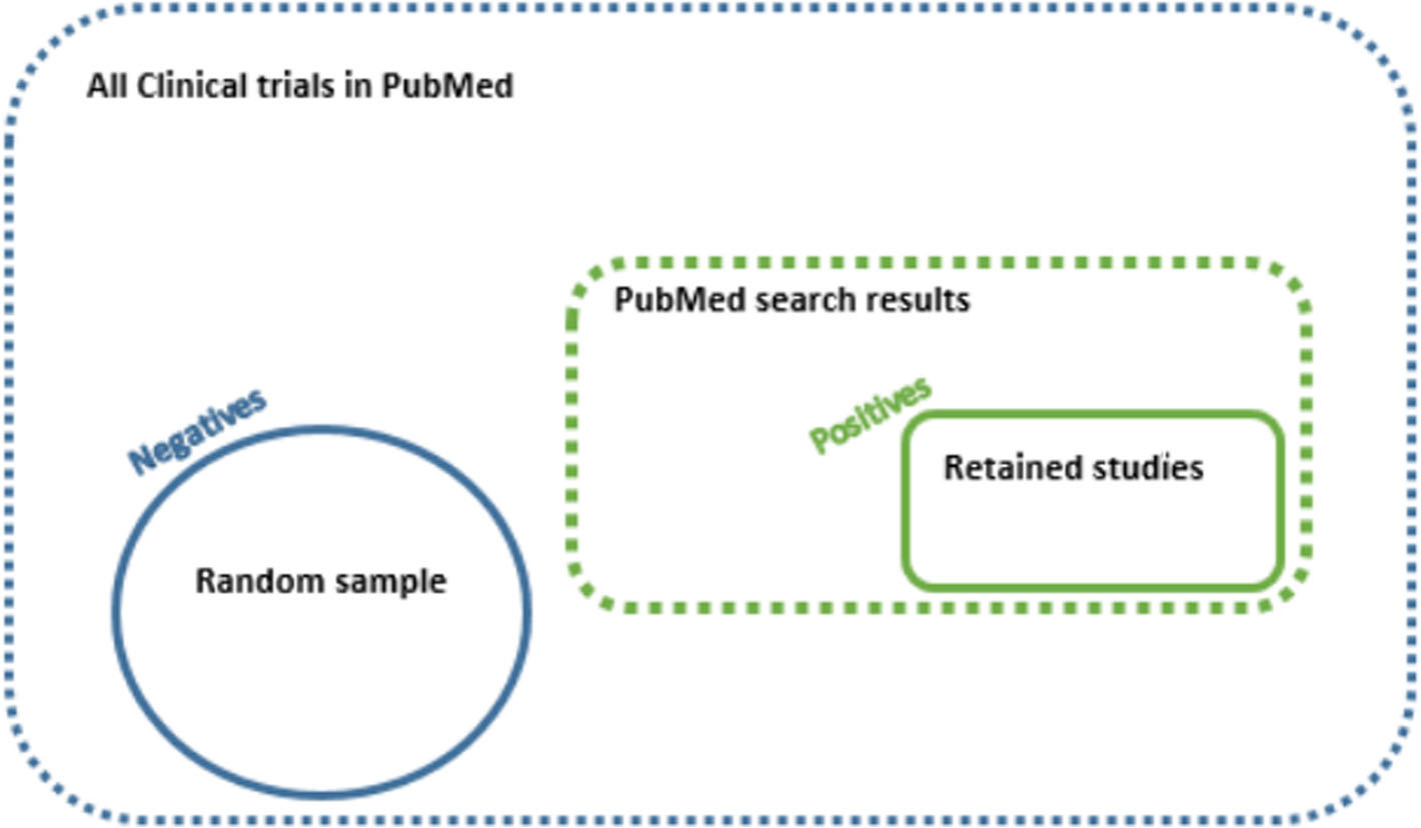
Building process of the training dataset. The positive citations are papers included in a systematic review*.* The negative citations are papers randomly selected from those completely off-topic. To identify positive citations, we recreate the input string in the PubMed database, using keywords and filters proposed in the original systematic review. Among retrieved records (dashed green line delimited region), we retain only papers finally included in the original systematic review (solid green line delimited region). On the other side, we randomly selected the negative citations (solid blue line delimited region) from Clinical Trial article type, according to PubMed filter, that were completely off-topic, i.e., by adding the Boolean operator NOT to the input string (region between green and blue dashed lines)

We considered the 14 SRs used and described in [14]. The training datasets contain the positive and negative citations retrieved from the PubMed database, where positives were the relevant papers finally included in each SR. To retrieve positive citations, for each SR, we ran the original search strings using identical keywords and filters. From the set of Clinical Trial article type (according to PubMed filter), we selected negative citations by adding the Boolean operator NOT to the original search string (see Fig. 1). The whole set of these negative citations was then sampled up to retain a minimum ratio of 1:20 (positives to negatives).

Further details on search strings and records retrieved in PubMed can be found in the supplementary material in [14]. The search date was the 18 July 2017. For each document (*n* = 7,494), information about the first author, year, title, and abstract were collected and included in the final dataset.

### Text pre-processing

We applied the following text pre-processing procedures to the title and abstract of each retrieved citation: each word was converted to lowercase, non-words were removed, stemming was applied, whitespaces were stripped away, and bi-grams were built and considered as a single token like a single word. The whole collection of tokens was finally used to get 14 document-term matrices (DTMs), one for each SR. The DTMs were initially filled by the term frequency (TF) weights, i.e., the simple counting number of each token in each document. The sparsity (i.e., the proportion of zero entries in the matrix) of the DTM was always about 99% (see Table 1). Term frequency-inverse document frequency (TF-IDF) [15] weights were used both for reducing the dimensionality of the DTMs by retaining the tokens ranked in the top 4% and as features used by the classifiers. The TF-IDF weights where applied to DTMs during each cross-validation (CV) step, accordingly to the same process described in [14].

**Table 1:** Characteristics of the document-term matrices (DTMs)

Systematic reviews	Documents	Tokens	*Non-zero* entries	*Zero* entries	Sparsity
Yang et al. 2014 [15]	418	61208	147445	25437499	0.99
Meng et al 2014 [16]	209	35821	73977	7412612	0.99
Segelov et al. 2014 [17]	413	58351	125027	23963936	0.99
Li et al. 2014 [18]	206	33851	68826	6904480	0.99
Lv et al. 2014 [19]	412	57485	138846	23544974	0.99
Wang et al. 2015 [20]	832	101418	288432	84091344	1.00
Zhou at al. 2014 [21]	209	33389	69854	6908447	0.99
Liu et al. 2014 [22]	623	88108	219258	54672026	1.00
Douxfils et al. 2014 [23]	413	58133	141721	23869208	0.99
Kourbeti et al. 2014 [24]	1675	187947	603479	314207746	1.00
Li et al. 2014 [25]	209	33653	69130	6964347	0.99
Cavender et al. 2014 [26]	414	59572	141105	24521703	0.99
Chatterjee et al. 2014 [27]	418	54458	130782	22632662	0.99
Funakoshi et al 2014 [28]	1043	131172	370385	136442011	1.00

For each, DTM reported the number of documents included (number of rows), the number of tokens included/computed within those documents (number of columns), the number of cells of the matrix which are filled with a 0 (zero), or a positive weight; the ratio of non-zero over the total ammount of entries (i.e., the sparsity) is also reported

### Chosen learners

We selected four commonly used classifiers in TM: support vector machines (SVMs) [16], k-nearest neighbor (k-NN) [17], random forests (RFs) [26], and elastic-net regularized generalized linear models (GLMNet) [28]. SVM and k-NN are among the most widely used MLTs in the text classification with low computational complexity [18]. Although computationally slower, RFs have also proved effective in textual data classification [19]. We selected GLMNets as benchmark linear model classifiers [20].

### Dealing with class imbalance

Random oversampling (ROS) and random undersampling (RUS) techniques were implemented to tackle the issue of class imbalance [10]. RUS removes the majority samples randomly from the training dataset to the desired ratio of the minority to majority classes. Since it reduces the dimensionality of the training dataset, it reduces the overall computational time as well, but there is no control over the information being removed from the dataset [10]. ROS adds the positive samples, i.e., the ones in the minority class, randomly in the dataset with replacement up to the desired minority to majority class ratio in the resulting dataset.

We included two different ratios for the balancing techniques: 50:50 and 35:65 (the minority to the majority). The standard ratio considered is the 50:50. On the other hand, we also examined the 35:65 ratio as suggested in [21].

### Analysis

The 20 modeling strategies resulting from any combination of MLTs (SVM, k-NN, RF, GLMNet), balancing techniques (RUS, ROS), and balancing ratios (50:50, 35:65) plus the ones resulting from the application of MLTs without any balancing technique were applied to the SRs reported in [14].

Fivefold CV was performed to train the classifier. The area under receiver operating characteristic curve (AUC-ROC) was calculated for each of the ten random combinations of the tunable parameters of the MLTs. The considered parameters were the number of variables randomly sampled as candidates for the trees to be used at each split for RF, the cost (C) of constraints violation for SVM, the regularization parameter (lambda) and the mixing parameter (alpha) for GLMNet, and the neighborhood size (k) for k-NN. The parameters with the best cross-validated AUC-ROC were finally selected.

**Fig. 2:**
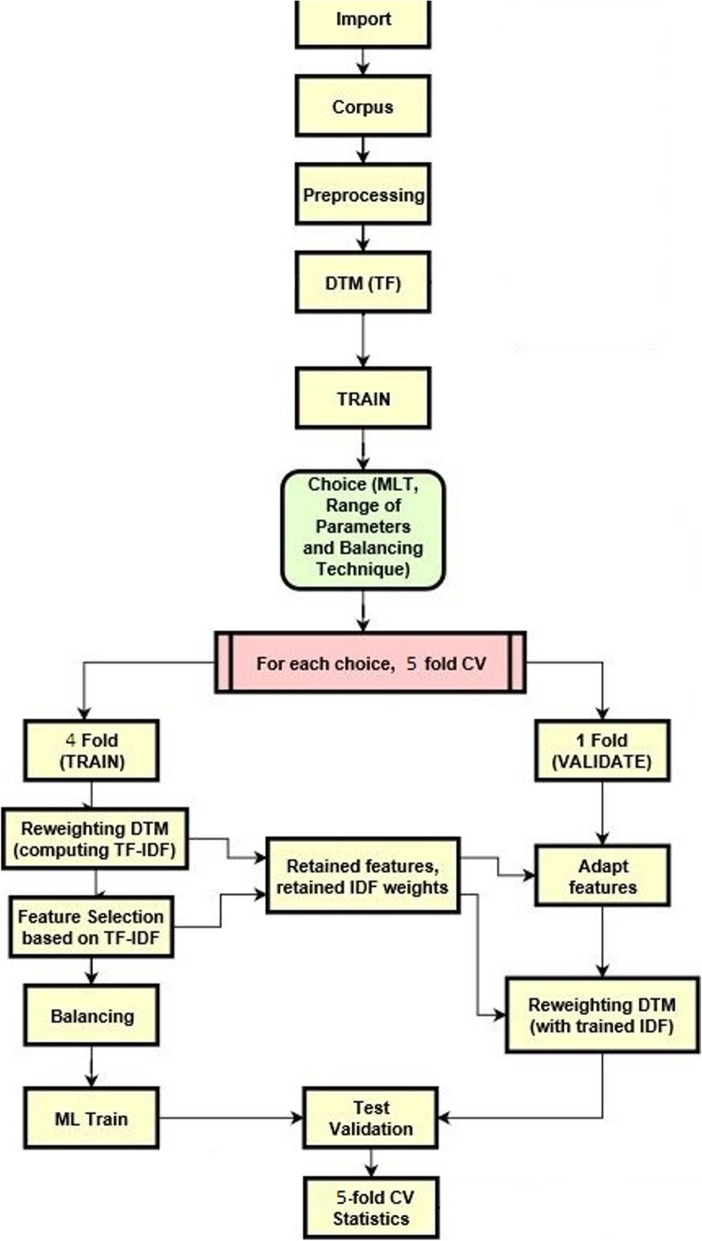
Computational plan. The set of documents for each systematic review considered was imported and converted into a corpus, preprocessed, and the corresponding document-term matrix (DTM) was created for the training. Next, for each combination of machine learning technique (MLT), each one of the corresponding ten randomly selected tuning parameters, and balancing technique adopted, the training was divided in fivefold for the cross-validation (CV) process. In each step of the CV, the DTM was rescaled to the term frequencies-inverse document frequencies (TF-IDF) weights (which are retained to rescale all the samples in the corresponding, i.e., the out-fold, test set). Next, the imbalance was treated with the selected algorithm, and the classifier was trained. Once the features in the test set were adapted to the training set, i.e., additional features were removed, missing ones were added with zero weight, and all of them were reordered accordingly; the trained model was applied to the test set to provide the statistics of interest

RUS and ROS techniques were applied to the training dataset. However, the validation data set was held out before using the text preprocessing and balancing techniques to avoid possible bias in the validation [22]. The whole process is represented in Fig. 2.

**Fig. 3:**
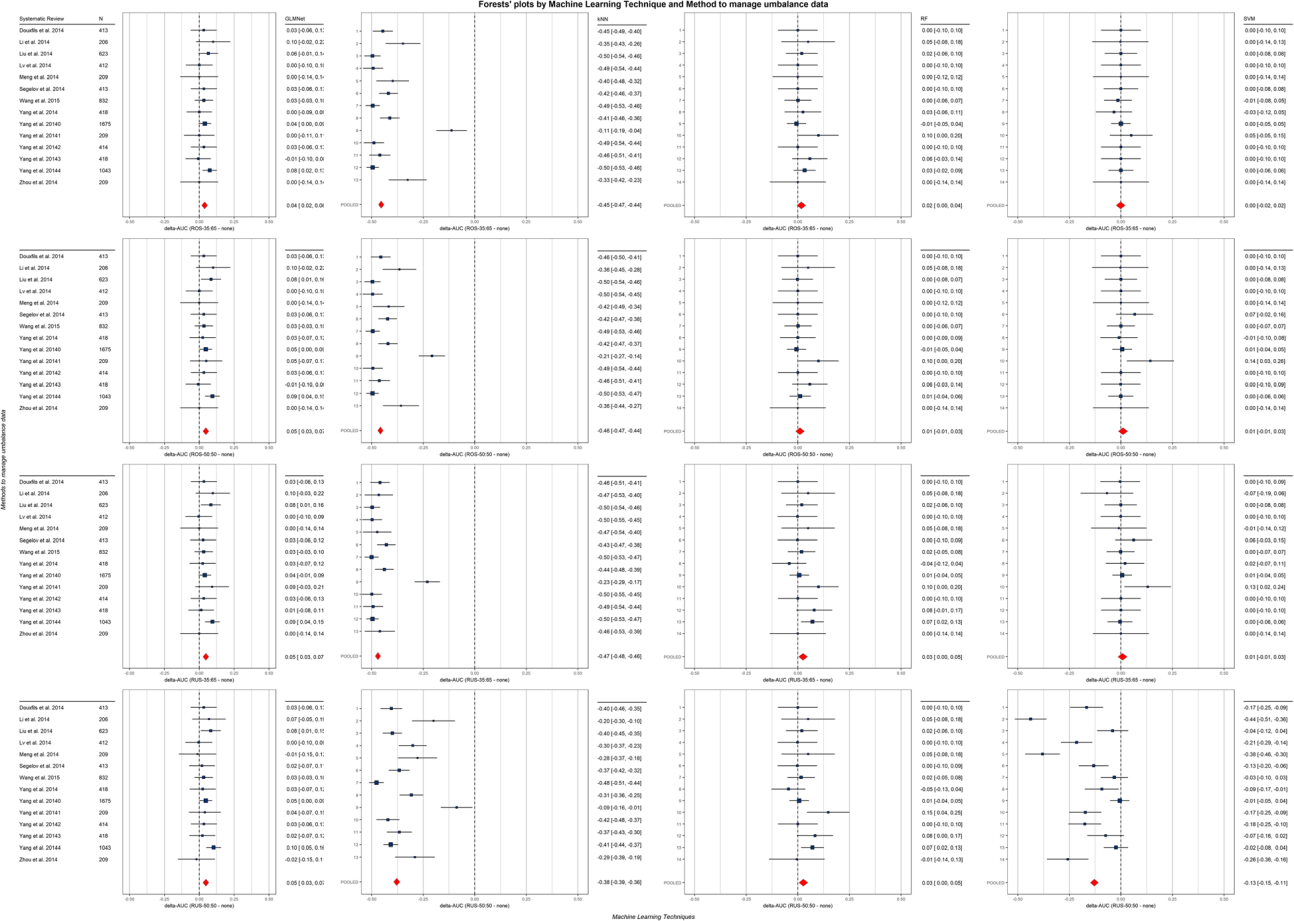
Forest plots of delta AUCs by balancing and machine learning techniques (MLTs). Forest plots that show differences in AUC (delta AUCs) between the AUCs obtained with each balancing technique (i.e., RUS-50:50, RUS-35:65, ROS-50:50, and ROS-35:65) and the AUC obtained without the application of any of them for each combination of MLT and systematic reviews. Red diamonds report to pooled results obtained with a by-MLT meta-analytic fixed-effect model. The first author and year of systematic review corresponding to each row of the forest plots are reported in the first column only, the MLTs are reported in the first row only, and the balancing techniques are reported in each forest plot’s *x*-axis label

To compare the results, separately for each MLT, we computed the within SR difference between the cross-validated AUC-ROC values resulting from the application of four balancing techniques (i.e., RUS and ROS both considering 50:50 and 35:65 possible balancing ratios) and the AUC-ROC resulting from the crude application of the MLT (i.e., by the “none” strategy to managing the unbalanced data). For all those delta AUCs, we computed 95% confidence intervals, estimated by the observed CV standard deviations and sample sizes. Next, we pooled the results by MLT using meta-analytic fixed-effect models. To evaluate the results, 16 forest plots were gridded together with MLTs by rows and balancing techniques by columns, in Fig. 3.

## Results

Table 2 reports cross-validated AUC-ROC values for each strategy, stratified by SR. In general, all the strategies achieved a very high cross-validated performance. Regarding the methods to handle class imbalance, ROS-50:50 and RUS-35:65 reported the best results. The application of no balancing technique resulted in a high performance only for the k-NN classifiers. Notably, for k-NN, the application of any method for class imbalance dramatically hampers its performance. A gain is observed for GLMnet and RF when coupled with a balancing technique. Conversely, no gain is observed for SVM.

**Table 2:** AUC-ROC values by combination of MLTs, balancing techniques and balancing ratios across 14 systematic reviews

MLT	Systematic review	Method for imbalance				
		None	ROS-35:65	ROS-50:50	RUS-35:65	RUS-50:50
GLMNet	Cavender et al. 2014 [26]	0.9667	*1*	*1*	0.9988	1
	Chatterjee et al. 2014 [27]	0.9738	0.9667	0.9667	0.9875	*0.9963*
	Douxfils et al. 2014 [23]	0.9667	0.9988	0.9988	*1*	0.9988
	Funakoshi et al 2014 [28]	0.8851	0.9602	*0.9799*	0.9794	0.9885
	Kourbeti et al. 2014 [24]	0.9518	0.9921	*0.9991*	0.9918	*0.9991*
	Li et al. 2014 [18]	0.9	*1*	*1*	0.9975	0.97
	Li et al. 2014 [25]	0.8975	0.8975	0.9475	*0.99*	0.9375
	Liu et al. 2014 [22]	0.915	0.98	*1*	0.9983	0.9975
	Lv et al. 2014 [19]	*1*	*1*	*1*	0.9963	0.9963
	Meng et al 2014 [16]	*1*	*1*	*1*	*1*	0.9875
	Segelov et al. 2014 [17]	0.9667	*1*	0.9988	0.995	0.9863
	Wang et al. 2015 [20]	0.9667	*1*	*1*	0.9988	0.9988
	Yang et al. 2014 [15]	0.975	0.975	*1*	*1*	*1*
	Zhou at al. 2014 [21]	*1*	1	*1*	*1*	0.98
k-nearest neighbors	Cavender et al. 2014 [26]	*1*	0.5113	0.5063	0.5013	0.5792
	Chatterjee et al. 2014 [27]	*0.9988*	0.5388	0.5363	0.5063	0.6333
	Douxfils et al. 2014 [23]	*0.9667*	0.5213	0.5113	0.5075	0.5625
	Funakoshi et al 2014 [28]	*0.9955*	0.5005	0.5	0.5	0.5885
	Kourbeti et al. 2014 [24]	NA	NA	NA	0.5	0.5661
	Li et al. 2014 [18]	*0.9775*	0.63	0.6125	0.5125	0.7775
	Li et al. 2014 [25]	*0.7975*	0.685	0.59	0.5675	0.71
	Liu et al. 2014 [22]	*0.9975*	0.5017	0.5017	0.5	0.5983
	Lv et al. 2014 [19]	*1*	0.5075	0.505	0.5025	0.6996
	Meng et al 2014 [16]	*0.9875*	0.59	0.57	0.515	0.71
	Segelov et al. 2014 [17]	*0.9283*	0.51	0.5063	0.5	0.5625
	Wang et al. 2015 [20]	*1*	0.5056	0.5056	0.5	0.5237
	Yang et al. 2014 [15]	*0.9404*	0.5288	0.52	0.5025	0.6333
	Zhou at al. 2014 [21]	*1*	0.675	0.6425	0.54	0.71
Random forest	Cavender et al. 2014 [26]	*1*	*1*	*1*	*1*	*1*
	Chatterjee et al. 2014 [27]	0.9167	0.975	0.975	0.9963	*1*
	Douxfils et al. 2014 [23]	*1*	*1*	*1*	*1*	*1*
	Funakoshi et al 2014 [28]	0.9184	0.9517	0.9299	*0.9895*	*0.9895*
	Kourbeti et al. 2014 [24]	0.9918	0.9854	0.9854	*0.9988*	0.9984
	Li et al. 2014 [18]	0.95	*1*	*1*	*1*	*1*
	Li et al. 2014 [25]	0.8	0.9	0.9	0.9	*0.9475*
	Liu et al. 2014 [22]	0.98	*0.9992*	0.9783	*0.9992*	*0.9992*
	Lv et al. 2014 [19]	*1*	*1*	*1*	0.9988	0.9988
	Meng et al 2014 [16]	0.95	0.95	0.95	*1*	*1*
	Segelov et al. 2014 [17]	*0.9988*	*0.9988*	*0.9988*	0.9975	0.9963
	Wang et al. 2015 [20]	0.9815	0.9821	0.9827	*0.9994*	0.9975
	Yang et al. 2014 [15]	0.95	*0.975*	0.95	0.9083	0.9046
	Zhou at al. 2014 [21]	*1*	*1*	*1*	*1*	0.995
Support vector machines	Cavender et al. 2014 [26]	*1*	*1*	*1*	*1*	0.825
	Chatterjee et al. 2014 [27]	*1*	*1*	0.9988	*1*	0.9263
	Douxfils et al. 2014 [23]	*1*	*1*	*1*	0.9963	0.8338
	Funakoshi et al 2014 [28]	*0.999*	*0.999*	0.9985	0.9945	0.975
	Kourbeti et al. 2014 [24]	0.9927	0.9927	*0.9991*	0.9988	0.9875
	Li et al. 2014 [18]	*1*	0.9975	0.9975	0.9325	0.5625
	Li et al. 2014 [25]	0.85	0.9	*0.9925*	0.98	0.6775
	Liu et al. 2014 [22]	*1*	*1*	*1*	0.9992	0.96
	Lv et al. 2014 [19]	*1*	*1*	*1*	0.9988	0.785
	Meng et al 2014 [16]	*1*	*1*	*1*	0.99	0.62
	Segelov et al. 2014 [17]	0.9333	0.9333	*1*	0.995	0.8013
	Wang et al. 2015 [20]	*1*	0.9857	*1*	0.9988	0.9681
	Yang et al. 2014 [15]	0.975	0.9417	0.9654	*0.995*	0.8825
	Zhou at al. 2014 [21]	*1*	*1*	*1*	*1*	0.7425

In italics are the best value(s) by row

*AUC-ROC* area under the receiver operator characteristic curve, *ROS* random oversampling, *RUS* random undersampling, *RF* random forest, *k-NN* k-nearest neighbors, *SVM* support vector machines, *GLMNet* elastic-net regularized generalized linear model

Meta-analytic analyses (see Fig. 3) show a significant improvement of the GLMNet classifier while using any strategy to manage the imbalance (minimum delta AUC of + 0.4 with [+ 0.2, + 0.6] 95% CI, reached using ROS-35:65). Regarding the application of strategies in combination with k-NN, all of them drastically and significantly hamper the performance of the classifier in comparison with the use of the k-NN alone (maximum delta AUC of − 0.38 with [− 0.39, − 0.36] 95% CI reached using RUS-50:50). About the RF classifier, the worst performance was reached using ROS-50:50 which is the only case the RF did not show a significant improvement (delta AUC + 0.01 with [− 0.01, + 0.03] 95% CI); in all the other cases, the improvements were significant. Last, the use of an SVM in combination with strategies to manage the imbalance shows no clear pattern in the performance, i.e., using RUS-50:50, the performance decreases significantly (delta AUC − 0.13 with [− 0.15, − 0.11] 95% CI); ROS-35:65 does not seem to have any effect (delta AUC 0.00 with [− 0.02, + 0.02] 95% CI); for both ROS-50:50 and RUS-35:56, the performance improves in the same way (delta AUC 0.01 with [− 0.01, + 0.03] 95% CI), though not significantly.

## Discussion

Application of MLTs in TM has proven to be a potential model to automatize the literature search from online databases [1–5]. Although it is difficult to establish any overall conclusions about best approaches, it is clear that efficiencies and reductions in workload are potentially achievable [6].

This study compares different combinations of MLTs and pre-processing approaches to deal with the imbalance in text classification as part of the screening stage of an SR. The aim of the proposed approach is to allow researchers to make comprehensive SRs, by extending existing literature searches from PubMed to other repositories such as ClinicalTrials.gov, where documents with a comparable word charactezisation could be accurately identified by the classifier trained on PubMed, as illustrated in [14]. Thus, for real-world applications, researchers must conduct the search string on citational databases, make the selection of studies to include in the SR, and add negative operator to the same search string to retrieve the negative citations. Next, they can use the information retrieved from the selected studies to train a ML classifier to apply on the corpus of the trials retrieved from ClinicalTrials.gov.

Regardless of the balancing techniques applied, all the MLTs considered in the present work have shown the potential to be used for the literature search from the online databases with AUC-ROCs across the MLTs (excluding k-NN) ranging prevalently above 90%.

Among study findings, the resampling pre-processing approach showed a slight improvement in the performance of the MLTs. ROS-50:50 and RUS-35:65 techniques showed the best results in general. Consistent with the literature, the use of k-NN does not seem to require any approach for imbalance [23]. On the other hand, for straightforward computational reasons directly related to the decrease in the sample size of the original dataset, the use of RUS 35:65 may be preferred. Moreover, k-NN showed unstable results when data had been balanced using whatever technique. It is also worth noting that k-NN-based algorithms returned an error, with no results, three times out of the 70 applications, while no other combination of MLT and pre-processing method encountered any errors. The problem occurred only in the SR of Kourbeti [24] which is the one with the highest number of records (75 positives and 1600 negatives), and only in combination with one of the two ROS techniques or when no technique was applied to handle unbalanced data, i.e., when the dimensionality does not decrease. The issue is known (see for instance the discussion in https://github.com/topepo/caret/issues/582) when using the caret R interface to MLT algorithms, and manual tuning of the neighborhood size could be a remedy [25].

According to the literature, the performance of various MLTs was found sensitive to the application of approaches for imbalanced data [11, 26]. For example, SVM with different kernels (linear, radial, polynomial, and sigmoid kernels) was analysed on a genomics biomedical text corpus using resampling techniques and reported that normalized linear and sigmoid kernels and the RUS technique outperformed the other approaches tested [27]. SVM and k-NN were also found sensitive to the class imbalance in the supervised sentiment classification [26]. Addition of cost-sensitive learning and threshold control has been reported to intensify the training process for models such as SVM and artificial neural network, and it might provide some gains for validation performances, not confirmed in the test results [28].

However, the high performance of MLTs in general and when no balancing techniques were applied are not in contrast with the literature. The main reason could be that each classifier is already showing good performance without the application of methods to handle unbalanced data, and there is no much scope left for the improvement. A possible explanation for such a good performance lies in the type of the training set and features, where positives and negatives are well-separated by design, and based on search strings performing word comparison into the metadata of the documents [14]. Nevertheless, the observed small relative gain in performance (around 1%) may translate into a significant absolute improvement depending on the intended use of the classifier (i.e., an application on textual repositories with millions of entries).

Study findings suggest that there is not an outperforming strategy to recommend as a convenient standard. However, the combination of SVM and RUS-35:65 may be suggested when the preference is for a fast algorithm with stable results and low computational complexity related to the sample size reduction.

## Limitations

Other approaches to handle unbalanced data could also be investigated, such as the algorithmic or the ensemble ones. Also, we decided to embrace the data-driven philosophy of ML and compare the different methods without any a priori choice and manual tuning of the specific hyper-parameter for each technique. This is with the final aim of obtaining reliable and not analyst-dependent results.

## Conclusions

Resampling techniques slightly improved the performance of the investigated machine learning techniques. From a computational perspective, random undersampling 35:65 may be preferred.

## Data Availability

Original data are publicly available, and the manuscript contains the description on how to retrieve them. Visit https://github.com/UBESP-DCTV/costumer for further information.

## Abbreviations

AUC-ROC: Area under receiver operating characteristic curve
CV: Cross-validation
DTM: Document-term matrix
GLMNet: Generalized linear model net
iDF: Inverse document frequency
k-NN: k-nearest neighbors
MLT: Machine learning technique
RF: Random forest
ROS: Random oversampling
RUS: Random undersampling
SR: Systematic review
SVM: Support vector machine
TF: Term frequency
TM: Text mining
